# Manufacture and Characterization of Recycled Polypropylene and Olive Pits Biocomposites

**DOI:** 10.3390/polym14194206

**Published:** 2022-10-07

**Authors:** Sofía Jurado-Contreras, Francisco J. Navas-Martos, José A. Rodríguez-Liébana, Alberto J. Moya, M. Dolores La Rubia

**Affiliations:** 1 Department of Chemical, Environmental and Materials Engineering, Campus Las Lagunillas, University of Jaén, 23071 Jaén, Spain; 2 Andaltec Technological Centre, Ampliación Polígono Industrial Cañada de la Fuente, C/Vilches 34, 23600 Martos, Spain; 3 University Institute for Research in Olive Grove and Olive Oil (INUO), Campus Las Lagunillas, University of Jaén, 23071 Jaén, Spain

**Keywords:** olive pits, biocomposites, recycled polypropylene, MAPP, mechanical properties

## Abstract

The present work studies the use of olive pit (OP) as a reinforcement in the manufacture of composites based on a post-consumer recycled polypropylene (rPP). In this way, it is feasible to provide added value from olive pits, a by-product resulting from the olive industry operations, while promoting the circular economy and reducing the use of fossil-based polymers. For this purpose, suitable samples were manufactured using 25 wt% and 40 wt% of OP. Additionally, the effect of incorporating additives was studied: (a) a process control additive (PA), and (b) a coupling agent of maleic anhydride grafted polypropylene (MAPP). The results showed an improvement in Young’s and flexural modulus with the OP addition. The incorporation of PA did not present any significant improvement in the properties of the materials, nevertheless it facilitated the biocomposite manufacturing process. As for the coupling agent, it significantly improved the mechanical properties, achieving the best results with the addition of the two types of additives and 40 wt% of OP. Moreover, the thermal properties were maintained, and there was an increase in crystallinity in all composites compared to rPP. According to the results of the fracture surface analysis, the coupling agent improves reinforcement-polymer matrix cohesion.

## 1. Introduction

The circular economy emerges as an effective tool to face the economic mechanisms and development processes implemented so far, which have led to the ecological depletion of the planet [1]. Polymer materials have represented a breakthrough for society, allowing the production of an endless number of low-cost and highly useful products. However, their inert nature and their resistance to biodegradation can generate a serious environmental problem derived from the management of their waste. At present, the disposal of polymer waste in controlled landfills does not guarantee an adequate procedure from the point of view of sustainability. In addition, other alternatives for managing this waste, such as mechanical recycling and incineration, are not always adequate solutions for the problems it poses. Therefore, the development of biocomposite materials represents an effective option to adequately manage this plastic waste [2]. That is why, among the actions contemplated, in the circular economy, is the ability to reuse polymeric materials and the manufacture of biocomposites to reduce environmental impact [1]. The Circular Economy Action Plan adopted in 2020 by European Economic and Social Committee highlights the use of biodegradable plastics or plastics with bio-based raw materials as one of the new challenges in terms of sustainability [3].

Currently, composites reinforced with by-products from the agricultural sector are considered a potential substitute for conventional polymer materials. By valorizing these agro-wastes, they become resources with high added value, which comply with the concept of circular economy [4]. There are numerous studies based on the incorporation of natural fibers from this sector in polymeric matrix: flax [5], hemp [6], jute [7] and sisal [8,9], among others.

Another possible way of reinforcing polymer matrix is the incorporation of residues from the fruit industry, such as hazelnut [10], walnut and almond [11] or peanut [12] shells. Núñez-Decap et al. (2021) studied the use of peach and cherry pit powder to manufacture polypropylene-based composite materials. Their results showed that the incorporation of peach pit particles, with respect to other studies performed with wood fibers, leads to a significant improvement in mechanical and physical properties [13]. Wechsler et al. (2019) carried out the incorporation of peach pit at 50 and 60 wt% for the manufacture of polypropylene-based composites, and compared their properties with those of a composite material formed with 60 wt% of pine wood fiber into the same polymer matrix. They observed a decrease in the mechanical properties of the peach pit-based composites. Such a trend can be attributed to poor cohesion of reinforcement and polymer matrix [14].

**Figure 1:**
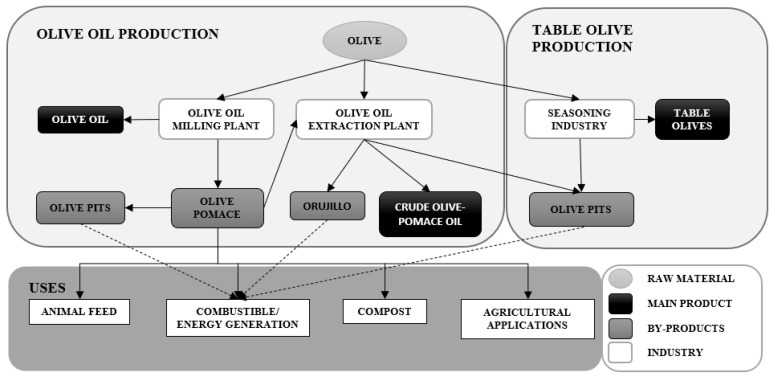
Simplified scheme of the processes involved in the production of olive oil and its by-products.

Internationally, olive groves occupy a total of 11.5 million hectares, representing 1% of the arable land of the planet. A large amount of waste is generated, 69.2% of which is currently not used [15]. When it comes to olive pits (OP), production in Europe is around 6,800,000 tons per year [16]. So far, its main route of valorization is its use as biofuel, due to its high energy content, generating ashes after combustion as a waste [17,18]. Research is currently being carried out on other ways of valorization: (a) in biorefinery, due to its high carbohydrate content [19], and (b) its incorporation as part of composite materials, such as the materials with which chipboard panels are manufactured [20]. Figure 1 shows a general overview of those processes involved in obtaining OP, as well as some of the feasible ways to valorize them.

In recent years, studies have been carried out for the incorporation of olive pit for manufacture of composite materials using different types of polymer matrices. Hamida et al. (2015) studied the incorporation of different weight proportions of OP powder for reinforcement a polystyrene matrix, observing a decrease in mechanical properties with the percentage of reinforcement, due to the hydrophilic nature of pits, which causes swelling of the polystyrene matrix [21]. Koutsomitopoulou et al. (2014), carried out the study of the influence of the addition of OP, of different particle sizes, into a polymer matrix of polylactic acid (PLA). It was observed that with any percentage of pits there was an increase in Young’s modulus, but a decrease in flexural strength [22]. Naghmouchi et al. (2015), carried out two studies on the incorporation of OP and wood fibers into a polymer matrix. They specifically analyzed the influence of incorporating one or both of the reinforcement into a polypropylene matrix, as well as the effect of incorporating a coupling agent. Composite materials made using this coupling agent showed a considerable improvement in terms of mechanical properties compared to mixtures that did not contain this additive. Similarly, it was found that the addition of fibers involved an increase in terms of mechanical properties with respect to those composites based on olive pit powder [23,24].

In this work, both the effect of the rPP matrix substitution ratio by OP (25 and 40 wt%) and the effect of the incorporation of two additives (PA and MAPP) were studied with the aim of analyzing the feasibility of incorporating OP for the manufacture of biocomposites as a potential solution for the sustainable management of this industrial waste [25], since being organic and biodegradable contributes to improving the environmental impact [26].

## 2. Materials and Methods

### 2.1. Conditioning and Characterization of Olive Pits

OP obtained from the olive milling systems were used as a reinforcement of the polymer matrix. They were previously conditioned for their suitable introduction into the polymer matrix. Several rounds of washing were carried out to remove the remains of soil and pomace. After the washing cycles, OP were dried at 40 °C in an oven for 24 h in order to remove moisture. Once OP were dried, they were adequately milled using a cryogenic milling system (RETSCH Ultra-Centrifugal Mill ZM 200, Haan, Germany), obtaining the OP sample with a suitable particle size. The milled OP were then put back into the oven at 30 °C until use to prevent them from acquiring moisture. The final particle size distribution of the resulting OP was determined using a Malvern Mastersizer 2000 equipment. Obtaining the values of d_10_, d_50_ and d_90_ which mean that 10%, 50% and 90% of the sample is smaller that this size value, respectively.

The moisture and ash content of the OP were determined after the milling process, using the Technical Association of Pulp and Paper Industry methods [27] (TAPPI T 12 os-75 and TAPPI T 15 os-58, respectively). The determination of the chemical composition in terms of cellulose, hemicellulose and lignin content was carried out following the methodology proposed by Browning [28], obtaining the carbohydrate content (D-glucose, D-xylose and L-arabinose) in a High-Performance Liquid Chromatography (HPLC) system and using X-ray diffraction (XRD) (Empyrean equipment with a PIXcel-3D detector from PANalytical, Malvern, United Kingdom) in the 2 theta range from 10 to 60° with a step size of 0.02. The identification of the chemical bonds of the different component of the OP sample was performed by Attenuated Total Reflectance-Fourier Transform Infrared spectroscopy ATR-FT-IR (Vertex 70 Bruker equipment, Billerica, MA, USA). The scans were recorded in the frequency range 4000–400 cm^−1^. The morphology of the OP before and after milling process was observed using a JEOL SM 840 (Tokyo, Japan) field emission scanning electron microscope (SEM).

### 2.2. Preparation of Biocomposite

**Figure 2:**
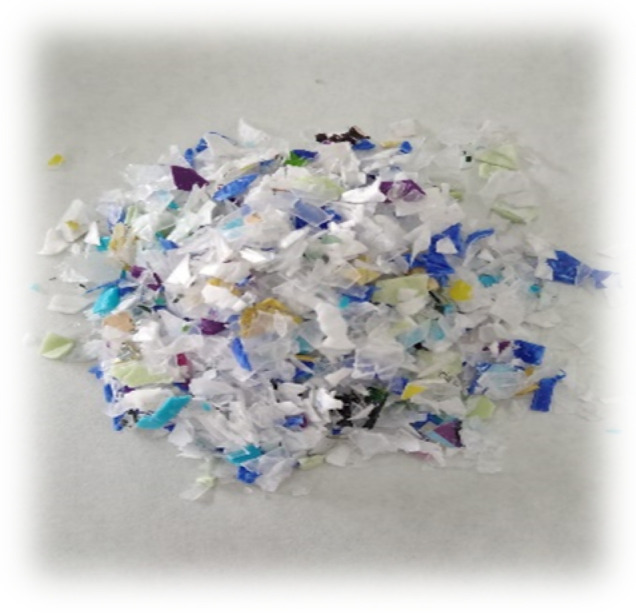
Recycled polypropylene.

Recycled polypropylene (ACTECO, Alicante, Spain) in the form of flakes with different shades was used as polymer matrix (Figure 2). A PA based on a fatty acid ester (BYK-Chemie GmbH, Offenbach am Main, Germany) was used in powder form. The MAPP used was initially in the form of pellets and is based on a polypropylene copolymer with a high level of maleic anhydride grafting (Exxon, Irving, TX, USA).

The incorporation of the additives, (PA) at 1.5 wt% and (MAPP) at 4 wt%, was carried out in two stages. Firstly, the effect produced by the addition of PA was studied, and secondly, both additives (PA and MAPP) were added in the manufacture process of the composites.

Prior to the preparation of the composites, the moisture of the OP sample was removed by drying in an oven for 24 h at 40 °C. Details of the different samples obtained are presented in Table 1. A Rehoscam internal Mixer system (Scamex, Crosne, France) was used for compounding. Four batches of 150 g each were prepared. After the compounding procedure, the resulting solid mass was shredded using a WSGM-250 milling system (J. Purchades, Madrid, Spain) to obtain the final material in the form of pellets.

### 2.3. Fabrication of Biocomposites Specimens

Once the pellets of each composite were obtained, the necessary test specimens were manufactured by injection molding technology to characterize the materials. Prior to the injection molding process, moisture was removed from the plastic material using a KKT 75 dryer (KOCH, Leopoldshöhe, Germany) at 60 °C for 2 h. The equipment used for the injection molding process was an ENGEL Victory 28 injection molding machine (Engel Holding GmbH, Schwertberg, Austria). The specimens manufactured were those whose dimensions complied with standards ISO 527-2, ISO 178 and 179-1, respectively [29,30,31].

### 2.4. Characterization of the Resulting Polymer Biocomposites

The composition of biocomposites was determined by XRD (Empyrean equipment with a PIXcel-3D detector from PANalytical) and recorded in the 2θ range from 10 to 60° with a step size of 0.02. In addition to the position of the peaks, their width was also determined. The half-width maximum (FWHM) values of samples were calculated to evaluate the size of the crystal. OriginPro 8.5.1 software was used for calculation [32].

The different chemical bonds of the biocomposite samples were determined by ATR-FTIR (Vertex 70 Bruker equipment, Billerica, MA, USA). The scans recorded in the frequency range 4000–400 cm^−1^. Tensile and flexural properties were determined using a universal testing machine 10KS (Tinius Olsen, Redhill, UK), according to ISO 527-2 and ISO 178 standards [29,30], respectively. A Charpy Impact Meter Izod IMPats 2281 (Metrotec, Lezo, Spain) was used to determine Charpy impact strength of every biocomposite according to ISO 179-1 standard [31]. A SEM coupled to Energy dispersive X-ray Spectroscopy (EDS) (JEOL SM 840, Tokyo, Japan) was used to perform microstructure observations of the fracture surface of the composite tested samples.

For the analysis of the thermal behavior, the DSC technique was carried out in a DSC 822e (Mettler Toledo, Barcelona, Spain). The heating rate used was 5 °C/min, during the heating mode in the range of 30 to 200 °C, maintaining this final temperature for three hours prior to the subsequent cooling to 30 °C. After analysis, the percentage of crystallinity was calculated using the following equation:

(1) $$W_{c\;}\left(\%\right)=\frac{\Delta H_{m}}{\Delta H_{\mathrm{mc}}}\;\times \;\frac{1}{f_{\mathrm{PP}}}\;\times \;100$$

where ΔH_m_ (J/g) corresponds to the enthalpy of fusion, ΔH_mc_ (J/g) is the enthalpy of fusion corresponding to a 100% crystalline polypropylene sample, 207 (J/g), [33] and f_pp_ corresponds to the fraction of rPP in the composite, in wt%.

## 3. Results and Discussion

### 3.1. Olive Pit Characterization

**Figure 3:**
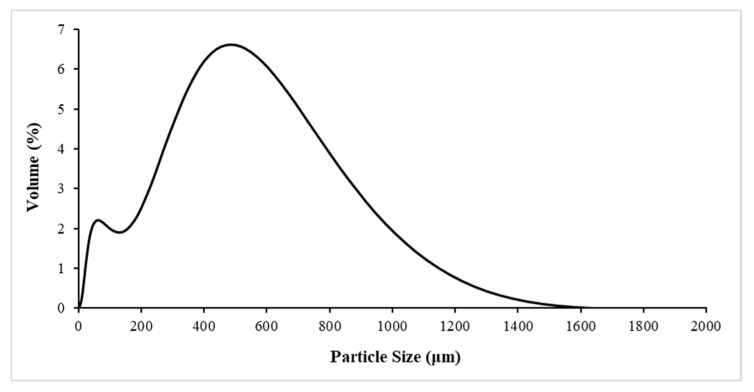
Particle size distribution of the OP milled sample.

The particle size distribution of the OP after the milling process is showed in Figure 3. The d_10_, d_50_ and d_90_ values shows in Table 2.

The values in Table 2 indicated that 10% of the OP sample was smaller than 40.3 µm, and the average particle size is 336 µm.

The results obtained, in terms of moisture, ash and OP composition, are shown in Table 3.

**Figure 4:**
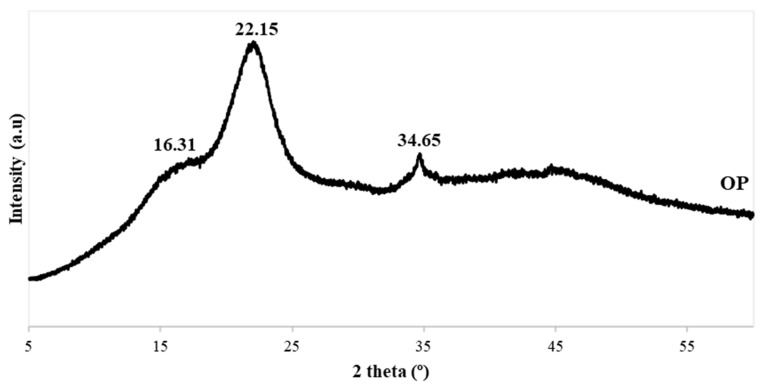
Olive pits XRD patterns.

Figure 4 shows the spectrum obtained from the XRD analysis of the pulverized OP. The graph presents three main peaks at approximately 16, 22 and 34.5°, which correspond to the characteristic peaks related to lignocellulosic materials [34]. These peaks come from the (110), (200) and (004) diffracting planes of cellulose crystals, respectively [35]. The reflection peak at 22° corresponds to the typical pattern of cellulose I, indicating the presence of cellulose [36].

**Figure 5:**
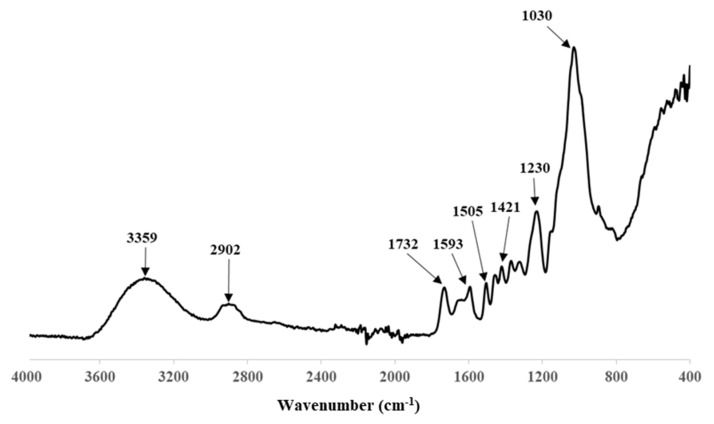
FT-IR performed on OP sample.

The FT-IR spectrum obtained from the OP sample is shown in Figure 5, where those functional groups typical of lignocellulosic materials can easily be observed. The bands indicate the presence of structures with C = C bonds corresponding to olefins (1700–1600 cm^−1^) and aromatic structures (1650–1500 cm^−1^), C-H (1480–1420 cm^−1^) and oxygenated groups: hydroxyl (1160–1000 cm^−1^), carbonyl (1770–1650 cm^−1^) and ether (1300–1000 cm^−1^) [37,38,39].

**Figure 6:**
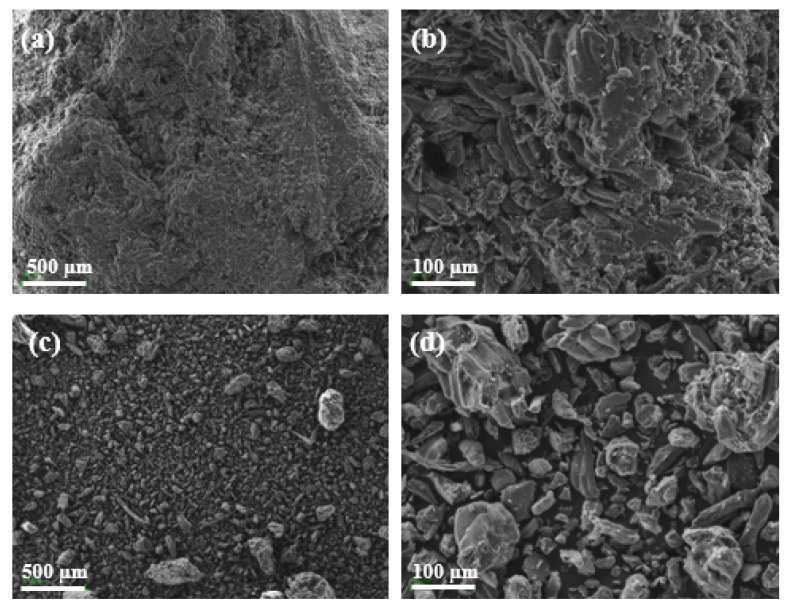
SEM images of OP samples: (**a**) not milled OP sample at 100×; (**b**) not milled OP sample at 500×; (**c**) milled OP sample at 100×; (**d**) milled OP sample at 500×.

Figure 6 shows the morphology of the OP samples before and after being milled at 100 and 500 magnification. Prior to being milled, the surface of the OP shows several pores and conductive tubes, characteristic of lignocellulose structures. After the milling process, the resulting particles presented spherical shapes according to the following figures. Figure 6c,d show the significant difference in terms of size distribution in the OP sample after and before being processed in the milling system. This indicates that this process affects the dimensional and topological properties of the OP resulting powder, and therefore also affects the particle size distribution which in turn affects the properties of the rPP/OP composites, as reported in other studies [22].

### 3.2. Mechanical Characterization of Biocomposites

**Figure 7:**
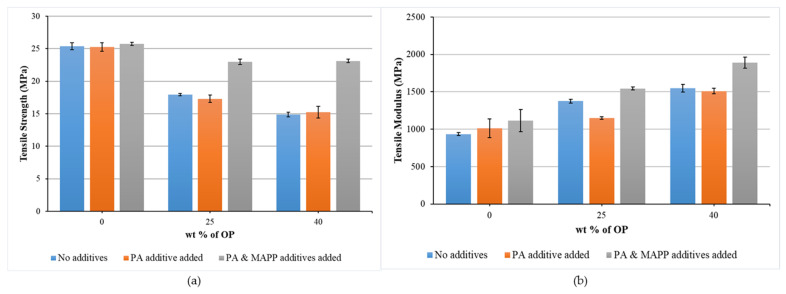
Tensile strength (**a**) and tensile modulus (**b**) of biocomposites as a function of percentage by weight of OP, considering materials without additives, adding PA and adding PA and MAPP, respectively.

Table 4 shows those parameters obtained from the tensile tests: maximum tensile stress (σ_m_), tensile stress at break (σ_b_), maximum tensile strain (ε_m_), strain at break (ε_b_) and Young’s modulus (E_t_). The tensile strength and Young’s modulus are shown in Figure 7.

The tensile strength (σ_m_) of the biocomposites decreased by 29.3% and 41.4% relative to that of rPP with the addition of 25% and 40% of OP, respectively. This decrease together with the one that takes place in terms of elongation at break of the composite is the result of the inefficient transfer from the polymer matrix to the reinforcement, due to the lack of interfacial bonding [22]. Other factors that can affect the process are the incompatibility between the polar reinforcement and the nonpolar polymer [40], as well as the poor dispersion of the reinforcement in rPP matrix, due to the interactions resulting from hydrogen bonding between OP particles [41]. Regarding the effect of the addition of the PA, σ_m_ decreased 31.5% and 39.7% relative to that of PA-rPP with the incorporation of 25% wt% and 40% wt% of OP, respectively. This decrease in properties is similar to the observed with rPP’s composites, so no significant influence is observed with the addition of PA in either of the two OP percentages used for the biocomposite manufacture. However, this additive is essential for the good processing of composites, since the introduction of reinforcements increases the viscosity of the resulting material [26]. On the contrary, the addition of MAPP does have a significant effect. The tensile strength of the biocomposites decreased by 10.8% and 10.2% relative to PA/MAPP-rPP when 25% wt% and 40% wt% of OP is added. These biocomposites reach tensile strength values higher than those of rPP. In addition, this improvement is more significant, reaching 14.88 MPa, when introducing up to 40 wt% OP to rPP matrix, and up to 23.13 MPa, when both additives are also added. The use of MAPP as a coupling agent has received attention for its effectiveness in improving the mechanical properties, especially tensile and flexural strength, of wood fiber-polypropylene composites [41,42]. The effectiveness of MAPP has been attributed to its ability to efficiently wet and disperse the reinforcement. More specifically, two types of mechanisms have been postulated for this effect: (a) the formation of ester bonds between the hydroxyl groups of the reinforcement and the anhydride carbonyl groups of the MAPP [43,44,45]; (b) the formation of an adhesive bridge between OP and polypropylene matrix, increasing the interfacial adhesion between the reinforcement and polypropylene matrix [44].

In the case of Young’s Modulus, the addition of the OP leads to an improvement in this property with respect to rPP, increasing up to 47.15% and 65.44%, when with 25 and 40 wt% OP is added, respectively. Similar behavior is observed with respect to PA-rPP and PA/MAPP-rPP with increases of 13.32% and 38.30% with 25 wt% OP and 49.02% and 69.49% with 40 wt% OP. The use of small, rigid filler particles into the polymer matrix can improve the Young’s modulus. This effect may be due to the increased area available for the OP particles to interaction with the matrix [46]. As for the effect of the addition of the PA, in the case of adding 40 wt% OP, no significant change was observed with respect to the 40OP-rPP. However, when adding 25 wt% OP and PA, the value decreased with respect to the same material without PA, although it remained above the rPP and PA-rPP values. The addition of MAPP increased Young’s modulus, this increase being more significant with a higher percentage of OP, going from 1116.56 MPa, for PA/MAPP-rPP, to 1890.81 Mpa, when adding the additives and 40 wt% OP, which means an improvement of 69.49% with respect PA/MAPP-rPP, but 102.21% compared with rPP. The composites with additives show E_t_ 12.12% and 22.22% higher than those without additives when 25 wt% and 40 wt% OP is added. Therefore, it can be concluded that the addition of olive pit can reinforce the rPP matrix, increasing the stiffness of the composite; and the addition of MAPP can improve the adhesion between the reinforcement and the polymer matrix. These results agree with what was reported by Tasdemir (2017), who studied the effect of the addition of olive pit and almond shell to a polymer matrix in terms of mechanical properties, obtaining the maximum increase with a reinforcement content of 40 wt% [40].

Table 5 shows those results obtained from flexural test: maximum flexural strength (σ_fm_), flexural strength at break (σ_fb_), maximum flexural strain (ε_fm_), flexural strain at break (ε_fb_) and flexural modulus (E_f_).

**Figure 8:**
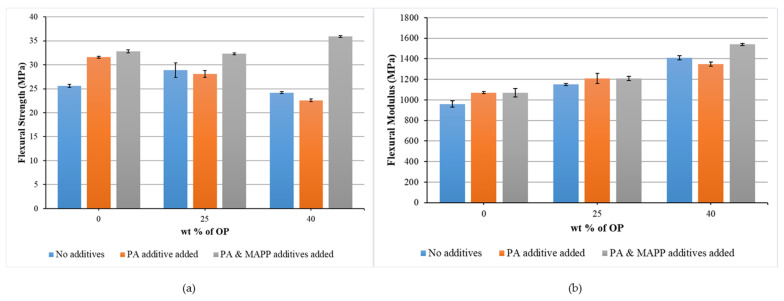
Flexural strength (**a**) and flexural modulus (**b**) of biocomposites as a function of percentage by weight of OP, considering materials without additives, adding PA and adding PA and MAPP, respectively.

Figure 8 presents the results of the flexural strength and modulus. As for σ_fb_, it increases with the introduction of 25 wt% OP, going from a value of 25.6 MPa of rPP to 28.9 MPa. However, it decreases when up to 40 wt% of reinforcement is added. This is also due to the lack of stress transfer between the polymer matrix and the reinforcement [47]. The addition of PA decreases the flexural strength of the biocomposites compared with PA-rPP matrix, which may be due to the low molecular weight of PA compared to rPP matrix and causing a plasticizing effect [48]. The addition of MAPP significantly improves the σ_fb_, being even higher when 40 wt% of OP is added, reaching an improvement of up to 9.45% and 40.23% compared to PA/MAPP-rPP and rPP, respectively.

Analyzing the values of E_f_ shown in Figure 8, as occurred with the values of the Young’s modulus, it increases with the introduction of the reinforcement, going from 960 MPa for rPP, up to 1150 MPa and 1410 MPa when 25 wt % and 40 wt% reinforcement is reached, respectively. These increases represent an improvement of 19.79 and 46.9% with respect to rPP. When adding 25 wt% OP, the additives do not provide a significant improvement. Nevertheless, when adding 40 wt% OP, the introduction of MAPP was a 9.2% increase compared to same material without additives, with improvements of 43.93% and 60.4% compared to PA/MAPP-rPP and rPP, respectively.

**Figure 9:**
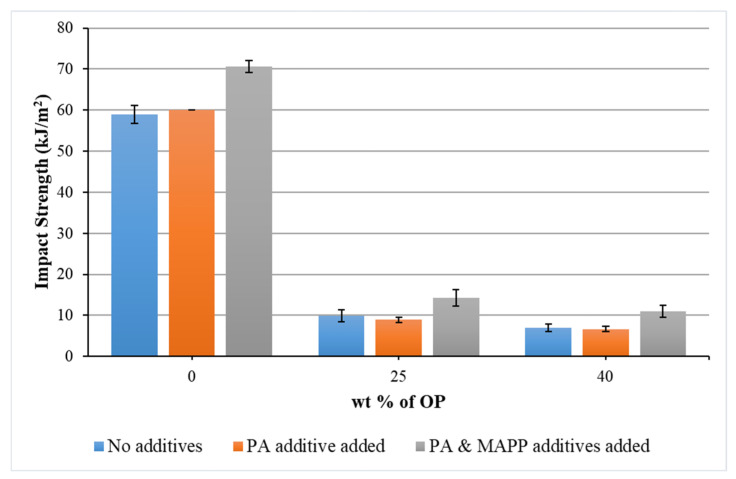
Results of the Charpy impact tests of biocomposites as a function of percentage by weight of OP, considering materials without additives, adding PA and adding PA and MAPP, respectively.

Figure 9 shows how the Charpy impact strength of the composite materials decreases with the introduction of OP, this decrease being greater as the weight percentage of OP increases. The usual mechanism of energy absorption, when performing the Charpy impact test, consists in crack propagation. A fracture initiates in areas where the stress concentration is located, probably in the interface between OP and polymer matrix. The reduction in impact strength with the reinforcement was also reported by Naghmouchi et al. (2015a) [23]. Nevertheless, the impact strength values obtained in those composites containing MAPP resulted to be the highest, so this additive clearly contributed to improving the bond between matrix and reinforcement.

### 3.3. Thermal Properties of Biocomposites

**Figure 10:**
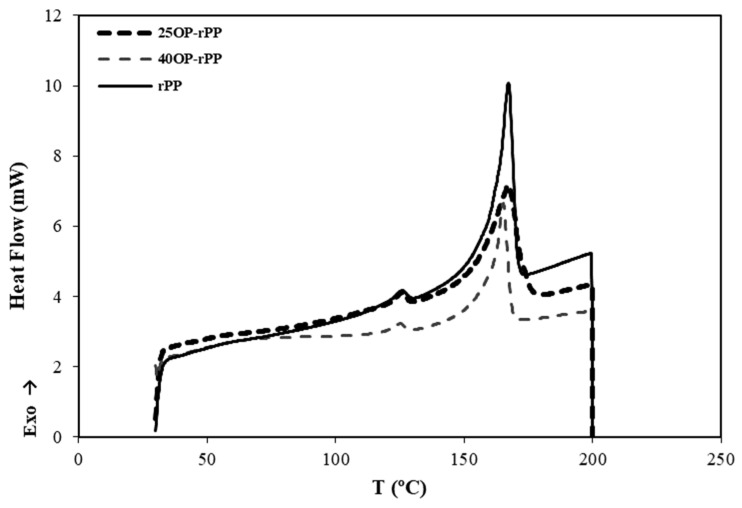
Thermograms resulting from the DSC performed on rPP and composites with 25 and 40 wt% OP without additives.

**Figure 11:**
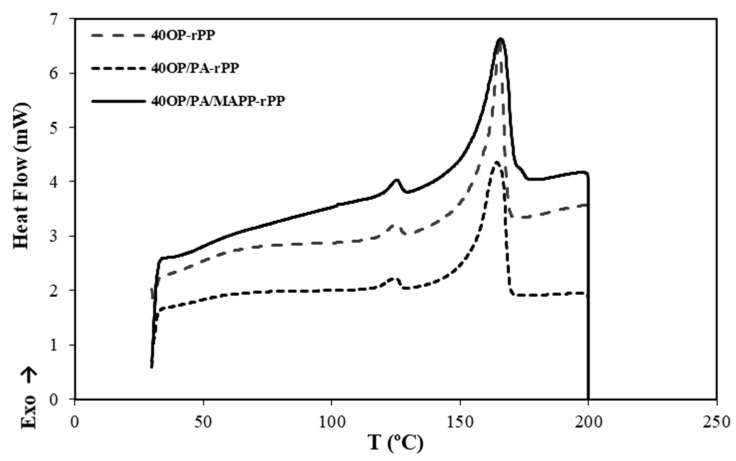
Thermograms resulting from the DSC performed on the composites reinforced with 40 wt% OP with and without additives.

DSC curves were obtained in order to study thermal capacity behavior of the OP-rPP biocomposites. The influence of the percentage of OP on the thermal properties is shown in Figure 10, where the thermograms resulting from the DSC performed at rPP and at the composites without additives are represented. Figure 11 shows the thermograms resulting from the DSC performed on the different composites with additives containing 40 wt% OP. Figure 10 and Figure 11 show two exothermic peaks: (a) between 160 °C and 180 °C, which indicate the melting temperature (T_m_); and (b) between 120 °C and 140 °C, corresponding to the recrystallization temperature (T_r_) [49].

Table 6 shows the results obtained for the Tr, Tm, enthalpy of fusion (ΔH_m_) and crystallinity (W_c_) of each polymer matrix and composite. The melting temperature values turned out to be very similar for all the material, so the incorporation of OP did not cause significant changes in their thermal behavior. Similar results were also reported by Techawinyutham et al., (2016), who did not observe changes in melting temperatures when lignocellulosic residue and MAPP additive were added to a polymer matrix [50]. Regarding crystallinity, all the composites presented a higher grade than rPP, which is in agreement with those results obtained in terms of mechanical properties and indicates the possibility of bond formation between rPP and OP. According to the results, the addition of 25%wt OP resulted in an 18.62% improvement in terms of Wc compared to rPP. The incorporation of PA decreased, when OP is added, the crystallinity, this decrease being even more significant when adding 40 wt% OP. Regarding the incorporation of MAPP additive, the crystallinity increases when incorporating 40 wt% OP due to the heterogeneous nucleation effect, since it performed as a nucleation agent [51]. Finally, the incorporation of both additives improves the crystallinity of the biocomposite containing 40 wt% OP by 12%, compared with rPP matrix with PA and MAPP.

### 3.4. X-ray Diffraction Analysis

**Figure 12:**
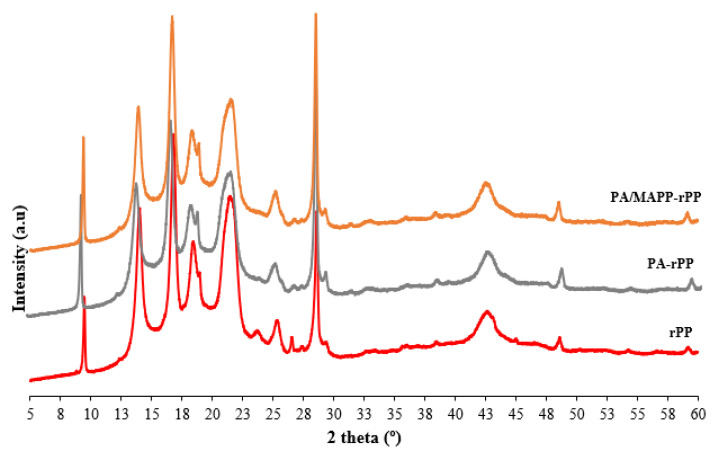
XRD pattern of rPP, PA-rPP and PA/MAPP-rPP.

**Figure 13:**
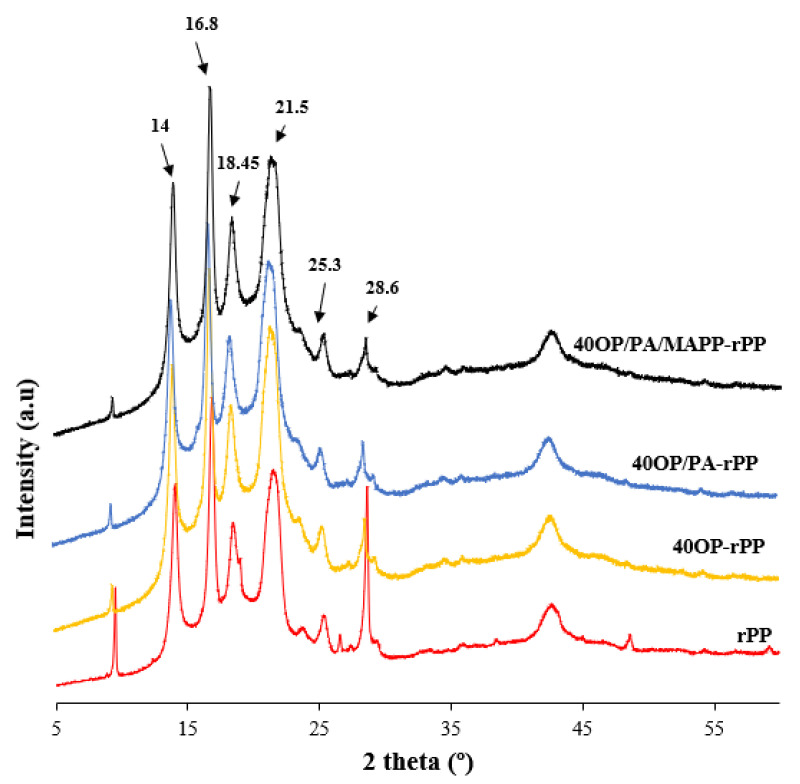
Diffraction diagram obtained as a result of the XRD analysis performed on the rPP and the different composites.

**Figure 14:**
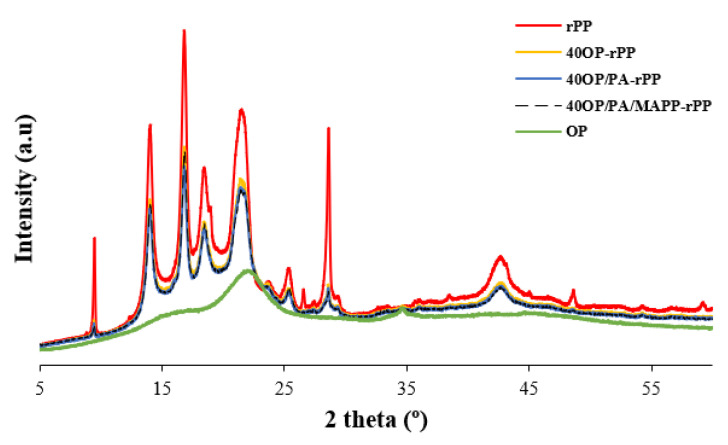
OP, rPP and biocomposites XRD-diffraction diagrams overlayed.

The XRD pattern of rPP, PA-rPP and PA/MAPP-rPP are presented in Figure 12. Figure 13 shows the XRD pattern of rPP and composites obtained by adding 40 wt% OP with and without additives. Figure 14, shows the overlapping of the XRD patter of the OP. The pattern corresponding to polypropylene presents three crystalline domains known as: α, β and δ. In the X-ray pattern corresponding to the rPP (Figure 12), the characteristic peaks of a polypropylene sample [-CH_2_ -CH(CH_3_)-], isotactic alpha form, are present. More specifically, five peaks can be observed at 2, 14, 17, 18.5, 21 and 28.5° [52,53]. The first group of four peaks corresponds to the typical crystallographic planes of pure polypropylene, (110), (040), (130), (111), reported by other authors [54]. After incorporation of the OP into the polymer matrix, there is a significant reduction in the intensity of all peaks. A displacement of the hump located in the 21–22° range is observed (Figure 14), which is indicative of the presence of the crystalline phase of cellulose I present in the OP [55]. The position of the rest of the peaks did not change in any of the manufactured composites, regardless of the addition of additives and the percentage of reinforcement (Figure 13). The same results were reported by other authors when incorporating lignocellulosic materials [56,57].

The results obtained for the samples with 40 wt% OP in the highest intensity peaks, responsible for the crystal structure and located at 14.1, 16.9, 18.6 and 21°, are shown in Table 7. The highest FWHM happens when adding OP, indicating a small crystal size, which is the reason for the increase in the regions indicated above in the XRD pattern. When PA and MAPP were added to the composites, a decrease in the size of the crystals was observed, with said size remaining even below those of rPP. A lower FWHM value is indicative of a higher homogeneity of the material [58].

### 3.5. Fourier Transform Infrared Spectroscopy

**Figure 15:**
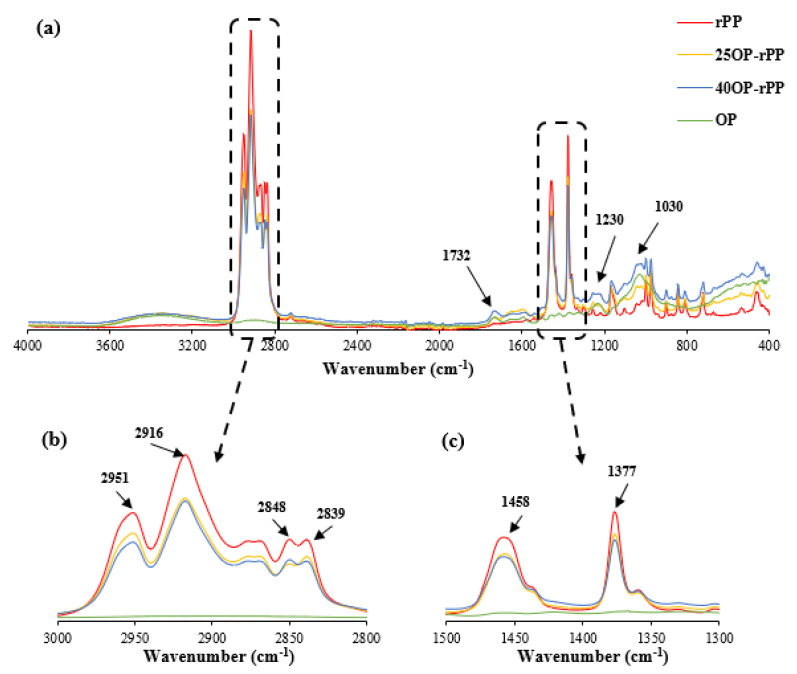
FT−IR Spectrum of rPP, OP and composites loaded with 25 and 40 wt% OP: (**a**) region: 400-4000 cm^−1^; (**b**) region: 2800-300 cm^−1^; (**c**) region: 1300–1500 cm^−1^.

Figure 15 shows the FT-IR spectrum of rPP, OP and the composites reinforced with 25 and 40 wt% OP. The spectrum corresponding to rPP shows the main peaks of a semi-crystalline polypropylene sample. Certain frequencies involve the CH_2_ and CH_3_ groups such as 2951 cm^−1^ (CH_3_), 2916 cm^−1^ (CH_2_), 2848 and 2839 cm^−1^ (CH_2_), 1458 cm^−1^ (CH_3_, CH_2_), 1377 cm^−1^ (CH_3_, CH_2_, CH), 1161 cm^−1^ (CH_3_, CH), 999 cm^−1^ (CH_3_, CH_2_, CH) and 972 cm^−1^ (CH_3_, CC) [59]. With the addition of OP particles, a decrease in the intensities of those peaks located at 2916 cm^−1^ (CH_2_), 2951 cm^−1^ (CH_3_), 2848 (CH_2_), 2839 cm^−1^ (CH_3_), 1458 cm^−1^ (CH_2_) and 1377 cm^−1^ (CH_3_), was observed. This is due to the OP particles physically affecting the carbon-hydrogen bonds [60]. The increase in a hump-shaped peak comprised in the spectral range between 3200 and 3600 cm^−1^, a region associated with the stretching of the -OH group, can be observed. This is indicative of the presence of the lignocellulosic material. The appearance of the peak located at 1230 cm^−1^ corresponding to the aromatic ethers, and a significant increase in the intensity of the peaks located at 1230 and 1030 cm^−1^, in 155 and 169%, respectively, in the samples of composites reinforced with a 40 wt% OP with respect to the rPP samples is indicative of the presence of lignocellulosic material [61].

**Figure 16:**
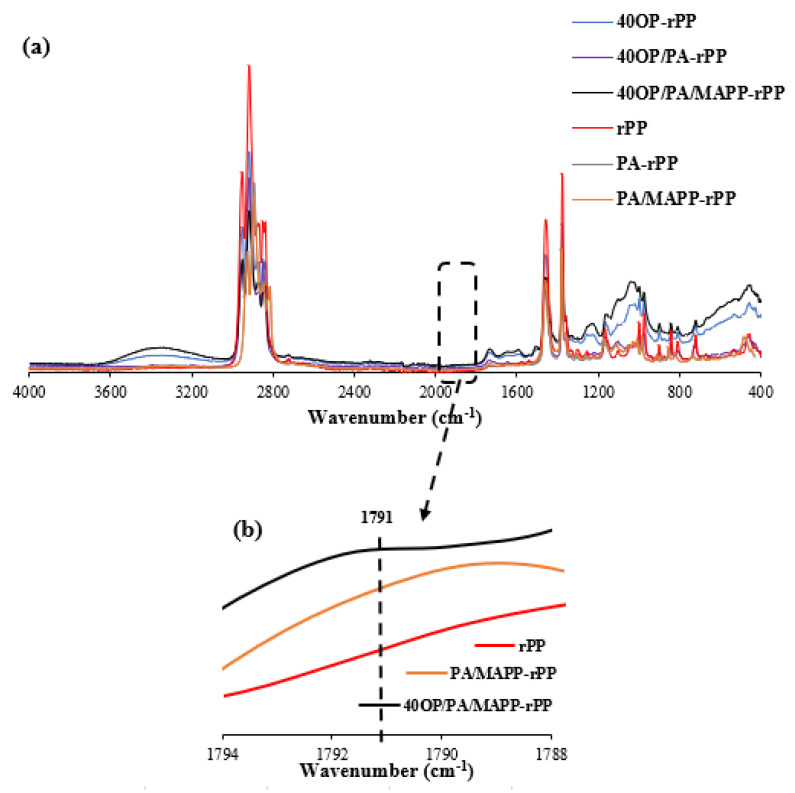
(**a**) FT−IR Spectra of composites loaded with 40 wt% OP with and without additives; (**b**) rPP, PA/MAPP-rPP and 40OP/PA/MAPP-rPP detailed in region between 1788–1794 cm^−1^.

The influence of the incorporation of additives is shown in the spectra of Figure 16. Regarding the MAPP influence, the presence of maleic anhydride is detected in the 1788–1794 cm^−1^ region. Figure 16b shown an increase in the characteristic band located near 1791 cm^−1^ is observed, which can be attributed to the =CO symmetric stretching of the anhydride functional groups grafted onto the polypropylene. This band is localized at a low frequency, indicating a low yield of anhydride grafting [62].

Furthermore, the -OH bond band located between 3100 and 3600 cm^−1^ increased with the addition of MAPP, possibly due to the hydroxyl group between the two hydrophobic structures [63].

**Figure 17:**
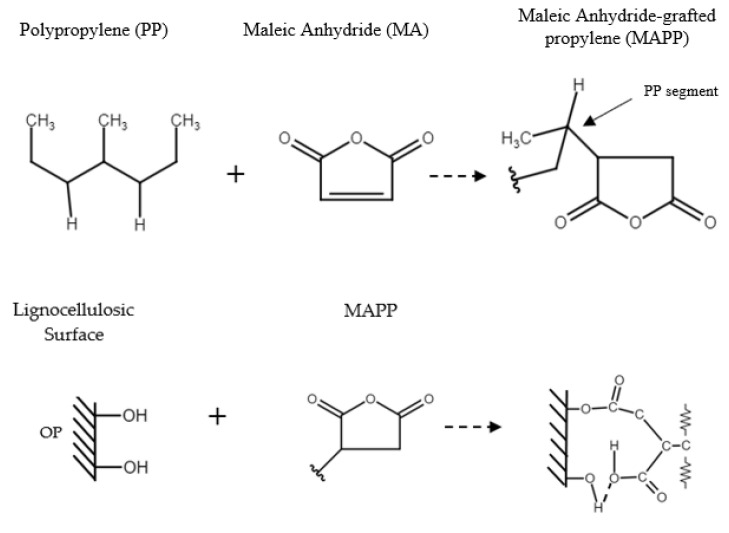
Mechanism of interaction between PP, MAPP and OP.

In Figure 17, the possible bonds between MAPP, OP and the polypropylene matrix [64] are shown. As can be seen, the hydroxyl groups on the OP surface can react with the MAPP anhydride group to form a new carbonyl group [65]. This effect can be seen in (Figure 15) in the intensity increase in the band attributed to the carbonyl functional groups between 1800 and 1600 cm^−1^, when MAPP is introduced into the bio-composite [63,65].

### 3.6. Morphological Analysis

**Figure 18:**
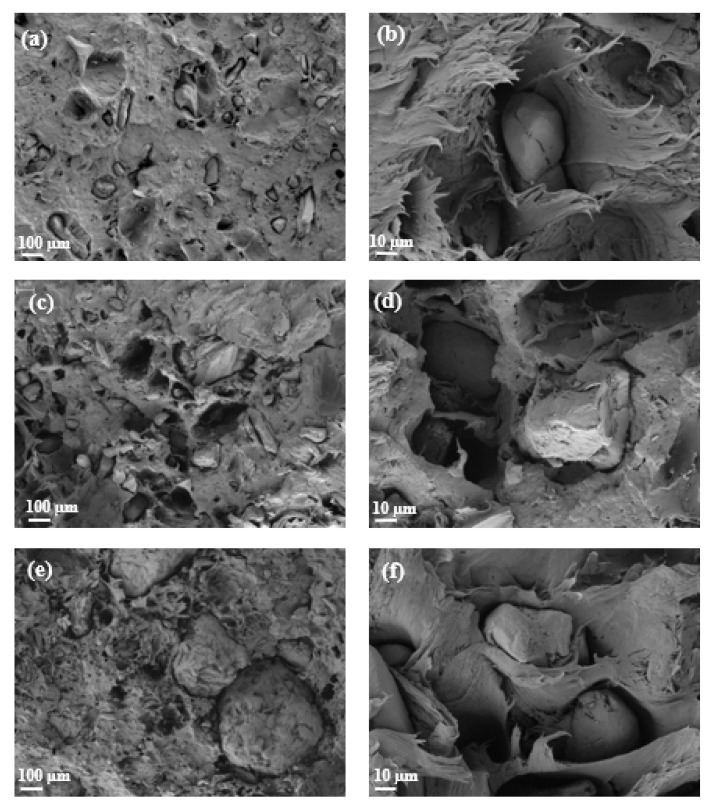
SEM micrographs of composites: (**a**) 25OP-rPP (500×); (**b**) 25OP-rPP (2000×); (**c**) 40OP-rPP (500×); (**d**) 40OP-rPP (2000×); (**e**) 40OP/PA-rPP (500×); (**f**) 40OP/PA-rPP (2000×).

Figure 18 shows the scanning electron micrographs obtained from fracture surface of those composite specimens subjected to the flexural test: 25OP-rPP, 40OP-rPP, 40OP/PA-rPP and 40OP/PA/MAPP-rPP samples. Two images of each sample were taken at different magnifications (500× and 2000×). When comparing the surfaces of Figure 18a,b, the change in increasing the percentage of reinforcement from 25 to 40 wt% and a greater presence of OP particles are observed. A greater number of voids generated by the OP particles, which is indicative of poor adhesion between the matrix and the reinforcement can also be observed [40].

To study the change with the incorporation of PA, Figure 18f which represent the fracture surface of those composites loaded with 40 wt% OP and this additive, were analyzed. A more homogeneous distribution of the reinforcement into the polymer matrix was also observed, however no change in the polymeric matrix and no improvement in cohesion were observed.

**Figure 19:**
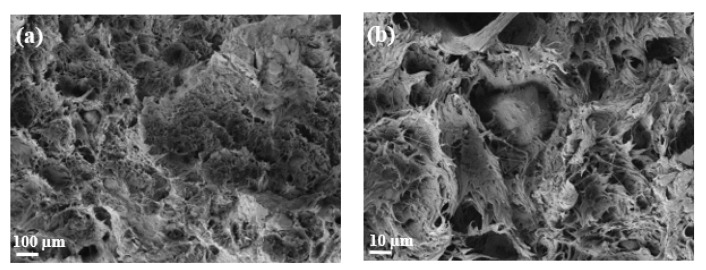
SEM micrographs of composites: (**a**) 40OP/PA/MAPP-rPP (500×); (**b**) 40OP/PA-rPP (2000×).

The addition of MAPP can be observed in Figure 19a,b. A stronger interaction between the OP particles and the matrix is observed. Therefore, the presence of MAPP into the composite material increases the adhesion between OP and rPP [66]. This phenomenon corroborates the effect of the coupling agent in terms of improving the mechanical properties [67]. Compatibilizers improve adhesion and affinity between both components, in addition to increasing the dispersion of the reinforcing particles in the matrix, while giving additional properties to the mixture [68,69].

## 4. Conclusions

The influence of the addition of different percentages of OP (25 and 40 wt%) as reinforcement of rPP was studied. In addition, the effect of the addition of PA and MAPP to the polymer matrix on the mechanical and thermal properties and the microstructure of the resulting polymer biocomposites was also analyzed. From the results obtained, the following conclusions were obtained:

The incorporation of OP, with no additives, decreases the tensile and flexural strength of resulting materials due to the lack of interfacial bonding between the reinforcement and the polymer matrix. The addition of PA does not influence these properties with respect to those biocomposites without PA. However, an increase was observed when incorporating MAPP. This improvement is due to an enhancement in the dispersion of the reinforcement into the polymer matrix. The Young’s modulus and flexural modulus increase significantly with the addition of reinforcement, increasing by 65.44% and 46.9%, respectively, when adding 40 wt% OP. The increase in these properties is even greater with the incorporation of MAPP, reaching E_t_ values 12.12% and—22.22% than those without additives when 25 wt% and 40 wt% OP is added. Similarly, E_f_ increased 5.22% and 9.22% compared to the composites without additives. It can be concluded that the OP can reinforce the polymer matrix in terms of stiffness, facilitating stress transfer, and MAPP can improve the interaction between the reinforcement and the polymer matrix. However, the impact strength of composites decreases with the presence of OP particles. The Charpy impact strength values improve using PA, although they remain below the rPP values.

Regarding the thermal properties, the values obtained for the melting temperature of the composites are similar, so the incorporation of the reinforcement does not cause any change in the thermal behavior. All the manufactured biocomposites present a higher Wc than rPP, which is consistent with the results in terms of mechanical properties and indicates the possibility of -H bond formation. The incorporation of MAPP increases the crystallinity for those composites with a reinforcement content of 40 wt% OP, which is due to the nucleation effect of the maleic anhydride-based additive.

The XRD results and the displacement observed in the FTIR spectra confirm the presence of the crystalline phase of the cellulose, typical of lignocellulosic residues. The analysis of the FTIR spectra shows the presence of maleic anhydride in the low absorption band located at 1788–1794 cm^−1^, which is indicative of a good -H bond between polymer matrix and OP. This corroborates the increase in mechanical properties and crystallinity. Decreasing FWHM values and SEM micrographs show that the addition of PA and MAPP improves the homogeneity and distribution of OP particles into the polymer matrix. SEM images show an increase in cohesion with the addition of MAPP.

Therefore, this research demonstrates that the incorporation of 40 wt% of OP into a rPP matrix and the use of PA and a coupling agent gives rise to resulting biocomposites with improved mechanical properties.

## Figures and Tables

**Table 1:** Composition of the different composites prepared.

Reference	rPP (wt%)	OP (wt%)	PA (wt%)	MAPP (wt%)
rPP	100.0	-	-	-
25OP-rPP	75.0	25.0	-	-
40OP-rPP	60.0	40.0	-	-
PA-rPP	100.0	-	1.5	-
25OP/PA-rPP	73.5	25.0	1.5	-
40OP/PA-rPP	58.5	40.0	1.5	-
PA/MAPP-rPP	100.0	-	1.5	4.0
25OP/PA/MAPP-rPP	69.5	25.0	1.5	4.0
40OP/PA/MAPP-rPP	54.5	40.0	1.5	4.0

**Table 2:** d_10_, d_50_ and d_90_ values obtained from the milled OP sample.

Raw Materials	d_10_ (µm)	d_50_ (µm)	d_90_ (µm)
OP	40.3	336	795

**Table 3:** Physico-chemical properties of OP.

Raw Materials	Moisture (wt%)	Ash (wt%)	Cellulose (wt%)	Hemicellulose (wt%)	Lignin (wt%)
OP	5.11	0.00	21.79	23.60	33.78

**Table 4:** Tensile properties of biocomposites and rPP.

Reference	σ_m_ (MPa)	σ_b_ (Mpa)	ε_m_ (%)	ε_b_ (%)	E_t_ (Mpa)
rPP	25.39 ± 0.56	18.87 ± 1.32	10.10 ± 0.40	17.25 ± 1.57	935.09 ± 18.77
25OP-rPP	17.95 ± 0.16	16.97 ± 0.34	6.32 ± 0.24	7.56 ± 0.19	1375.99 ± 26.35
40OP-rPP	14.88 ± 0.36	13.92 ± 0.33	4.95 ± 0.36	6.34 ± 0.49	1547.03 ± 50.02
PA-rPP	25.28 ± 0.65	19.72 ± 0.68	8.30 ± 0.15	30.86 ± 5.99	1012.54 ± 126.01
25OP/PA-rPP	17.31 ± 0.57	16.34 ± 0.52	6.24 ± 0.67	7.82 ± 0.75	1147.38 ± 16.62
40OP/PA-rPP	15.25 ± 0.91	14.63 ± 1.09	4.65 ± 0.16	5.42 ± 0.44	1508.91 ± 36.88
PA/MAPP-rPP	26.07 ± 0.25	20.70 ± 0.31	8.32 ± 0.28	24.36 ± 2.37	1115.53 ± 150.14
25OP/PA/MAPP-rPP	22.98 ± 0.41	22.43 ± 0.44	7.37 ± 0.28	8.18 ± 0.36	1542.78 ± 20.45
40OP/PA/MAPP-rPP	23.13 ± 0.28	22.72 ± 0.34	6.70 ± 0.20	7.55 ± 0.33	1890.81 ± 72.62

**Table 5:** Flexural properties of biocomposites.

Reference	σ_fm_ (MPa)	σf_b_ (MPa)	ε_fm_ (%)	ε_fb_ (%)	E_f_ (MPa)
rPP	29.50 ± 0.30	25.60 ± 0.30	5.00 ± 0.00	5.00 ± 0.00	960.00 ± 30.00
25OP-rPP	28.90 ± 1.50	28.90 ± 1.50	5.00 ± 0.00	5.00 ± 0.00	1150.00 ± 10.00
40OP-rPP	25.20 ± 0.20	24.20 ± 0.20	4.00 ± 0.10	4.90 ± 0.10	1410.00 ± 20.00
PA-rPP	31.60 ± 0.20	31.60 ± 0.20	4.90 ± 0.00	5.00 ± 0.10	1070.00 ± 40.00
25OP/PA-rPP	28.10 ± 0.70	28.10 ± 0.70	5.00 ± 0.00	5.00 ± 0.00	1210.00 ± 50.00
40OP/PA-rPP	22.90 ± 0.20	22.60 ± 0.30	4.20 ± 0.10	5.00 ± 0.00	1350.00 ± 20.00
PA/MAPP-rPP	32.90 ± 0.20	33.00 ± 0.30	4.90 ± 0.00	5.00 ± 0.10	1110.00 ± 40.00
25OP/PA/MAPP-rPP	32.30 ± 0.20	32.30 ± 0.20	5.00 ± 0.00	5.00 ± 0.00	1210.00 ± 20.00
40OP/PA/MAPP-rPP	35.90 ± 0.20	35.90 ± 0.20	5.00 ± 0.00	5.00 ± 0.00	1540.00 ± 10.00

**Table 6:** Values of the different thermal parameters obtained from the DSC curves of the rPP, PA-rPP, PA/MAPP-rPP and the different composites.

Reference	W_c_ (%)	T_r_ (°C)	T_m_ (°C)	ΔH_m_ (J/g)
rPP	22.72 ± 0.07	126.22 ± 0.23	168.41 ± 1.22	47.18 ± 0.15
25OP-rPP	27.92 ± 0.37	125.92 ± 0.09	167.74 ± 0.57	43.93 ± 0.57
40OP-rPP	26.20 ± 2.42	125.13 ± 0.07	165.44 ± 0.25	35.55 ± 3.00
PA-rPP	26.08 ± 0.91	125.68 ± 0.15	170.46 ± 0.73	55.87 ± 1.88
25OP/PA-rPP	26.73 ± 2.66	125.32 ± 0.02	166.69 ± 0.92	36.62 ± 4.06
40OP/PA-rPP	24.22 ± 0.09	125.29 ± 0.38	166.21 ± 1.75	29.44 ± 0.10
PA/MAPP-rPP	24.20 ± 0.76	125.80 ± 0.39	171.03 ± 1.06	48.54 ± 1.57
25OP/PA/MAPP-rPP	24.76 ± 0.36	126.21 ± 0.45	167.44 ± 0.17	36.14 ± 0.52
40OP/PA/MAPP-rPP	27.10 ± 1.84	125.98 ± 0.82	166.81 ± 0.94	28.5 ± 0.28

**Table 7:** FWHM values in the reflection peaks present in the XRD patterns of the biocomposites.

Reference	14°	17°	18.5°	21°
rPP	0.4952	0.4439	0.6860	1.1869
40OP-rPP	0.5084	0.4478	0.6440	1.2946
40OP/PA-rPP	0.4685	0.4414	0.6013	1.2167
40OP/PA/MAPP-rPP	0.4496	0.4293	0.5750	1.2678

## Data Availability

The data presented in this study are available on request from the corresponding author.

## References

[1] Bonnenfant C., Gontard N., Aouf C. (2022). Biobased and Biodegradable Polymers in a Circular Economy Context: Understanding Quercetin and Gallic Acid Impacts on PHBV Thermal Properties. Polym. Degrad. Stab..

[2] Moriana R. (2010). Development and Characterization of Fibered Biocomposites from Renewable Resources. Study of Its Degradation on Land. Ph.D. Thesis.

[3] European Commission (2020). Communication from the Commission to the Council.

[4] Ferri M., Vannini M., Ehrnell M., Eliasson L., Xanthakis E., Monari S., Sisti L., Marchese P., Celli A., Tassoni A. (2020). From Winery Waste to Bioactive Compounds and New Polymeric Biocomposites: A Contribution to the Circular Economy Concept. J. Adv. Res..

[5] Arbelaiz A., Fernández B., Valea A., Mondragon I. (2006). Mechanical Properties of Short Flax Fibre Bundle/Poly(Ε-Caprolactone) Composites: Influence of Matrix Modification and Fibre Content. Carbohydr. Polym..

[6] Bhowmik R., Das S., Mallick D., Gautam S.S. (2022). Predicting the Elastic Properties of Hemp Fiber—A Comparative Study on Different Polymer Composite. Mater. Today Proc..

[7] Kumar P., Allamraju K.V. (2021). Experimental Study and Characterization of Glass, Jute & Sisal Fiber Reinforced Polymer Matrix Composites. Mater. Today Proc..

[8] Kumar N., Grewal J.S., Kumar S., Kumar N., Kashyap K. (2021). Mechanical and Thermal Properties of Naoh Treated Sisal Natural Fiber Reinforced Polymer Composites: Barium Sulphate Used as Filler. Mater. Today Proc..

[9] Badyankal P.V., Manjunatha T.S., Vaggar G.B., Praveen K.C. (2021). Compression and Water Absorption Behaviour of Banana and Sisal Hybrid Fiber Polymer Composites. Mater. Today Proc..

[10] Balart J.F., Fombuena V., Fenollar O., Boronat T., Sánchez-Nacher L. (2016). Processing and Characterization of High Environmental Efficiency Composites Based on PLA and Hazelnut Shell Flour (HSF) with Biobased Plasticizers Derived from Epoxidized Linseed Oil (ELO). Compos. B Eng..

[11] Pirayesh H., Khanjanzadeh H., Salari A. (2013). Effect of Using Walnut/Almond Shells on the Physical, Mechanical Properties and Formaldehyde Emission of Particleboard. Compos. B Eng..

[12] Güler C., Büyüksarı Ü. (2011). Effect of Production Parameter on the Physical and Mechanical Properties of Particleboards Made from Peanut (*Arachis hypogaea* L.) Hull. BioResources.

[13] Núñez-Decap M., Wechsler-Pizarro A., Vidal-Vega M. (2021). Mechanical, Physical, Thermal and Morphological Properties of Polypropylene Composite Materials Developed with Particles of Peach and Cherry Stones. Sustain. Mater. Technol..

[14] Wechsler A., Molina J., Cayumil R., Núñez-Decap M., Ballerini-Arroyo A. (2019). Some Properties of Composite Panels Manufactured from Peach (*Prunus persica*) Pits and Polypropylene. Compos. B Eng..

[15] Marquina J., Colinet M.J., Pablo-Romero M.P. (2021). The Economic Value of Olive Sector Biomass for Thermal and Electrical Uses in Andalusia (Spain). Renew. Sustain. Energy Rev..

[16] Espadas-Aldana G., Guaygua-Amaguaña P., Vialle C., Belaud J., Evon P., Sablayrolles C. (2021). Life Cycle Assessment of Olive Pomace as a Reinforcement in Polypropylene and Polyethylene Biocomposite Materials: A New Perspective for the Valorization of this Agricultural By-Product. Coatings.

[17] González J.F., González-García C.M., Ramiro A., González J., Sabio E., Gañán J., Rodríguez M.A. (2004). Combustion Optimisation of Biomass Residue Pellets for Domestic Heating with a Mural Boiler. Biomass Bioenergy.

[18] Mami M., Mätzing H., Gehrmann H., Stapf D., Bolduan R., Lajili M. (2018). Investigation of the Olive Mill Solid Wastes Pellets Combustion in a Counter-Current Fixed Bed Reactor. Energies.

[19] Calvano C., Tamborrino A. (2022). Valorization of Olive By-Products: Innovative Strategies for Their Production, Treatment and Characterization. Foods.

[20] Elbir M., Moubarik A., Rakib E.M., Grimi N., Amhoud A., Miguel G., Hanine H., Artaud J., Vanloot P., Mbarki M. (2012). Valorization of Moroccan Olive Stones by Using It in Particleboard Panels. Maderas Ciencia y Tecnología.

[21] Hamida B., Ahmed M., Nadia N., Samira M. (2015). Mechanical Properties of Polystyrene/Olive Stone Flour Composites. Res. J. Pharm. Biol. Chem. Sci..

[22] Koutsomitopoulou A.F., Bénézet J.C., Bergeret A., Papanicolaou G.C. (2014). Preparation and Characterization of Olive Pit Powder as a Filler to PLA-Matrix Bio-Composites. Powder Technol..

[23] Naghmouchi I., Mutjé P., Boufi S. (2015). Olive Stones Flour as Reinforcement in Polypropylene Composites: A Step Forward in the Valorization of the Solid Waste from the Olive Oil Industry. Ind. Crops Prod..

[24] Naghmouchi I., Espinach F.X., Mutjé P., Boufi S. (2015). Polypropylene Composites Based on Lignocellulosic Fillers: How the Filler Morphology Affects the Composite Properties. Mater. Des..

[25] Valvez S., Maceiras A., Santos P., Reis P.N.B. (2021). Olive Stones as Filler for Polymer-Based Composites: A Review. Materials.

[26] La Mantia F., Dintcheva N.T., Morreale M., Vaca-Garcia C. (2004). Green Composites of Organic Materials and Recycled Post-Consumer Polyethylene. Polym. Int..

[27] (2018). Test Methods. Technical Associaton for the Pulp and Paper Industries.

[28] Browning B.L. (1967). Determination of lignin. Methods of Wood Chemistry.

[29] (2012). Plastics—Determination of Tensile Properties—Part 2: Test Conditions for Moulding and Extrusion Plastics (ISO 527-2:2012).

[30] (2020). Plastics—Determination of Flexural Properties (ISO 178:2019).

[31] (2011). Plastics—Determination of Charpy Impact Properties—Part 1: Non-Instrumented Impact Test (ISO 179-1:2000).

[32] Panigrahi H., Sreenath P.R., Bhowmick A.K., Kumar K.D. (2019). Unique Compatibilized Thermoplastic Elastomer from Polypropylene and Epichlorohydrin Rubber. Polymer.

[33] Kilic A., Jones K., Shim E., Pourdeyhimi B. (2015). Surface Crystallinity of Meltspun Isotactic Polypropylene Filaments. Macromol. Res..

[34] Reichert A.A., Ribas M., Escobar G., Augusto C., Fajardo A.R. (2020). Utilization of Pineapple Crown Fiber and Recycled Polypropylene for Production of Sustainable Composites. J. Renew. Mater..

[35] Correia C., Oliveira L., Valera T. (2017). The Influence of Bleached Jute Fiber Filler on the Properties of Vulcanized Natural Rubber. Mater. Res..

[36] Benyahia A., Merrouche A., Rahmouni Z., Mansour R., Serge W., Kouadri Z. (2014). Study of the Alkali Treatment Effect on the Mechanical Behavior of the Composite Unsaturated Polyester-Alfa Fibers. Mech. Ind..

[37] Belalia M., Bendjelloul M., Aziz A., Elandaloussi E. (2018). Surface Modification of Olive Stone Waste for Enhanced Sorption Properties of Cadmium and Lead Ions. Acta Chem. Iasi.

[38] Corzo M. (2004). El Hueso de Cereza, un Residuo Agroindustrial Objeto de Estudio para El Aprovechamiento en la Obtención De Carbonizados y Carbones Activados. Rev. Estud. Extrem..

[39] Hasanin M.S., Kassem N., Hassan M.L. (2021). Preparation and Characterization of Microcrystalline Cellulose from Olive Stones. Biomass Convers. Biorefin..

[40] Tasdemir M. (2017). Effects of Olive Pit and Almond Shell Powder on Polypropylene. Key Eng. Mater..

[41] Woodhams R.T., Thomas G., Rodgers D.K. (1984). Wood Fibers as Reinforcing Fillers for Polyolefins. Polym. Eng. Sci..

[42] Sanadi A.R., Young R.A., Clemons C., Rowell R.M. (1994). Recycled Newspaper Fibers as Reinforcing Fillers in Thermoplastics: Part I-Analysis of Tensile and Impact Properties in Polypropylene. J. Reinf. Plast. Compos.

[43] Kishi H., Yoshioka M., Yamanoi A., Shiraishi N. (1989). Composites of Wood and Polypropylene. Mokuzai Gakkaishi.

[44] Felix J.M., Gatenholm P. (1991). The Nature of Adhesion in Composites of Modified Cellulose Fibers and Polypropylene. J. Appl. Polym. Sci..

[45] Kazayawoko M., Matuana L.M., Balatinecz J.J. (1999). Surface Modification and Adhesion Mechanisms in Woodfiber-Polypropylene Composites. J. Mater. Sci..

[46] Essabir H., EL Achaby M., Hilali E.M., Bouhfid R., Qaiss A., Hilali E.M. (2015). Morphological, Structural, Thermal and Tensile Properties of High Density Polyethylene Composites Reinforced with Treated Argan Nut Shell Particles. J. Bionic Eng..

[47] Naghmouchi I., Mutjé P., Boufi S. (2014). Polyvinyl Chloride Composites Filled with Olive Stone Flour: Mechanical, Thermal, and Water Absorption Properties. J. Appl. Polym. Sci..

[48] Khalid M.S., Ali S., Abdullah L.C., Ratnam C.T., Choong S.Y.T. (2006). Effect of MAPP as Coupling Agent on the Mechanical Properties of Palm Fiber Empty Fruit Bunch and Cellulose Polypropylene Biocomposites. Int. J. Eng. Technol..

[49] Caldas V., Brown G.R., Nohr R.S., MacDonald J.G., Raboin L.E. (1994). The Structure of the Mesomorphic Phase of Quenched Isotactic Polypropylene. Polymer.

[50] Techawinyutham L., Frick A., Siengchin S. (2016). Polypropylene/Maleic Anhydride Grafted Polypropylene (MAgPP)/Coconut Fiber Composites. Adv. Mech. Eng..

[51] Meng-Heng W., Cheng-Chien W., Chuh-Yung C. (2020). Chemical Modification of Atactic Polypropylene and Its Applications as a Crystallinity Additive and Compatibility Agent. Polymer.

[52] Mora J.C., Esquivel M., Durán M., Zamora R. (2015). Obtaining and evaluation of polypropylene mixtures with banana rachis fibers (Musa AAA). Rev. Iberoam. Polím..

[53] Clack E.S., Mark J.E. (1999). Unit Cell Information on Some Important Polymers.

[54] Morales-Cepeda A.B., Ponce-Medina M.E., Salas-Papayanopolos H., Lozano T., Zamudio M., Lafleur L.G. (2015). Preparation and Characterization of Candelilla Fiber (*Euphorbia antisyphilitica*) and Its Reinforcing Effect in Polypropylene Composites. Cellulose.

[55] Panaitescu D.M., Vuluga Z., Ghiurea M., Iorga M., Nicolae C., Gabor R. (2015). Influence of Compatibilizing System on Morphology, Thermal and Mechanical Properties of High Flow Polypropylene Reinforced with Short Hemp Fibers. Compos. B Eng..

[56] Ryu Y.S., Lee J.H., Kim S.H. (2020). Efficacy of Alkyl Ketene Dimer Modified Microcrystalline Cellulose in Polypropylene Matrix. Polymer.

[57] Zhang Y.-C., Wu H.-Y., Qiu Y.-P. (2010). Morphology and Properties of Hybrid Composites Based on Polypropylene/Polylactic Acid Blend and Bamboo Fiber. Bioresour. Technol..

[58] Jain S., Goossens H., van Duin M., Lemstra P. (2005). Effect of in Situ Prepared Silica Nano-Particles on Non-Isothermal Crystallization of Polypropylene. Polymer.

[59] Andreassen E. (1999). Infrared and Raman Spectroscopy of Polypropylene.

[60] Gümüş B.E., Yağci Ö., Erdoğan D.C., Tasdemir M. (2019). Dynamical Mechanical Properties of Polypropylene Composites Filled with Olive Pit Particles. J. Test. Eval..

[61] Anukam A.I., Mamphweli S.N., Reddy P., Okoh O.O. (2016). Characterization and the Effect of Lignocellulosic Biomass Value Addition on Gasification Efficiency. Energy Explor. Exploit..

[62] Sclavons M., Laurent M., Devaux J., Carlier V. (2005). Maleic Anhydride-Grafted Polypropylene: FTIR Study of a Model Polymer Grafted by Ene-Reaction. Polymer.

[63] Ragunathan S., Zainal M., Kamarudin H., Sam S.T., Ismail H. (2017). The Effect of Polypropylene Maleic Anhydride on Polypropylene/(Recycled Acrylonitrile Butadiene Rubber)/(Sugarcane Bagasse) Composite. J. Vinyl Addit. Technol..

[64] Ndiaye D., Diop B., Thiandoume C., Fall P.A., Farota A.K., Tidjani, Dogan F. (2012). Morphology and Thermo Mechanical properties of wood polypropylene composites. Polypropylen.

[65] Minggang L., Xin W., Jie L., Tao T. (2014). Synergetic Effect of Epoxy Resin and Maleic Anhydride Grafted Polypropylene on Improving Mechanical Properties of Polypropylene/Short Carbon Fiber Composites. Compos. Part A Appl. Sci. Manuf..

[66] Arráez F.J., Ávila M., Arnal M.L., Müller A.J. (2018). Study of the Influence of Pro-Oxidant Additives During the Oxodegradation of Polypropylene and High Impact Polystyrene Films. Revista Latinoamericana de Metalurgia y Materiales.

[67] Zainal M., Aihsan M.Z., Mustafa W.A., Santiagoo R. (2018). Experimental Study on Thermal and Tensile Properties on Polypropylene Maleic Anhydride as a Compatibilizer in Polypropylene/Sugarcane Bagasse Composite. J. Adv. Res. Fluid Mech. Therm. Sci..

[68] Laske S., Kracalik M., Gschweitl M., Feuchter M., Maier G., Pinter G., Thomann R., Friesenbichler W., Langecker R. (2009). Estimation of Reinforcement in Compatibilized Polypropylene Nanocomposites by Extensional Rheology. J. Appl. Polym. Sci..

[69] Nosova N., Roiter Y., Samaryk V., Varvarenko S., Stetsyshyn Y., Minko S., Stamm M., Voronov S. (2004). Polypropylene Surface Peroxidation with Heterofunctional Polyperoxides. Macromol. Symp..

